# Efficient Aggregate Queries on Location Data with Confidentiality

**DOI:** 10.3390/s22134908

**Published:** 2022-06-29

**Authors:** Da Feng, Fucai Zhou, Qiang Wang, Qiyu Wu, Bao Li

**Affiliations:** Software College, Northeastern University, Shenyang 110000, China; dfengneu@gmail.com (D.F.); wangq3635@126.com (Q.W.); kathywuqy@gmail.com (Q.W.); libaoneu@gmail.com (B.L.)

**Keywords:** aggregate query, confidentiality, location data, private set intersection, Paillier cryptosystem

## Abstract

Location data have great value for facility location selection. Due to the privacy issues of both location data and user identities, a location service provider can not hand over the private location data to a business or a third party for analysis or reveal the location data for jointly running data analysis with a business. In this paper, we propose a newly constructed PSI filter that can help the two parties privately find the data corresponding to the items in the intersection without any computations and, subsequently, we give the PSI filter generation protocol. We utilize it to construct three types of aggregate protocols for facility location selection with confidentiality. Then we propose a ciphertext matrix compressing method, making one block of cipher contain lots of plaintext data while keeping the homomorphic property valid. This method can efficiently further reduce the computation/communication cost of the query process—the improved query protocol utilizing the ciphertext matrix compressing method is given followed. We show the correctness and privacy of the proposed query protocols. The theoretical analysis of computation/communication overhead shows that our proposed query protocols are efficient both in computation and communication and the experimental results of the efficiency tests show the practicality of the protocols.

## 1. Introduction

With the widespread popularity of electronic devices with GPS functions and thanks to advances in information and communication technologies, location-based services (LBS) have developed significantly, and location service providers (LSP), such as Google Maps and FourSquare, already own a very large amount of user location data, which are of great commercial value in facility location selection. LSPs seek to find ways to provide location data aggregate analysis services to other businesses. Businesses would also like to pay for this kind of service for location selection because an appropriate location can bring much more economic interest than an ordinary location. Specifically, for example, a bank plans to open a branch, and it has several candidate addresses. “Which is the best location” is a puzzle for the bank because it does not have the location data of its users, so the bank seeks the help of the LSPs. Even in some public sectors (communal facilities) where economic interests are not the core issue, for businesses that define “value” as being used by more people or making something convenient for more people, a proper location makes the public facilities more valuable. The desire of the LSP and the business to cooperation raises a problem that the LSP can not simply send location data to the business or a third party for analyzing or reveal the location data for jointly running a data analysis query with the business due to privacy issues in both terms of law and ethics. From the above points of view, how to run a two-party privacy-preserving query between an LSP and a business is a meaningful topic.

The selection of a facility location among alternative locations has been widely studied, which is regarded as a multi-criteria decision-making problem, including both quantitative and qualitative criteria [1]. The location selection query is to choose a location with maximum influence for a new facility [2]. We mainly focus on three types of objectives [3] for location selection—(1) maximizing the number of users attracted (Maximum is not appropriate for some special businesses such as express. Instead, they may pursue sharing workload equally for each facility. For this objective, the RNNC query is still applicable.); (2) minimizing the average distance from all users to their nearest facilities; (3) minimizing the maximum distance from all users to their nearest facilities. The above selection indicators correspond to three types of aggregate queries, respectively, as follows:

(1) Reverse Nearest Neighbor Cardinality (RNNC) query. The RNN query is a very common query for location selection. In our two-party privacy-preserving query scenario, for a (public) set of existing facilities and given query locations, the RNN query computes all the users that are closer to each given query location than to any other facilities. Considering the first objective above, our RNNC query only needs to return the set of the cardinalities of the RNN.
(2) Average Distance (AVGD) query. The average distance represents the mean value of the distances between all users and their nearest facilities. The average distance is valuable information for the client to minimize is to maximize user benefit. The AVGD query result measures the impact of a candidate location of the new facility on all users, which can help a business such as a supermarket to decide where to put a new branch store in order to maximize the benefit to its customers. In short, the minimized average distance corresponds to the most valuable location.
(3) Maximum Distance (MAXD) query. The maximum distance represents the maximum value of the distances between all users and their nearest facilities. It takes the worst case as a selection indicator, i.e., after running several MAXD queries returning the maximum distances corresponding to different candidate locations, the business can compare those and choose the location which has the smallest one to maximize the benefits to the most “inconvenient” user.

In practice, for multiple alternative locations, a business can run one of these three types of queries according to the chosen criterion for each location, then decide which location is more beneficial to it.

In this paper, we use “server (S)” to replace the LSP that provides services of aggregate queries and use “client (C)” to replace the business that needs location data analyses. Consistently, we use $U_{s}/U_{c}$ to represent S/C’s user list. We continue to focus on the practical settings described in [3]: (1) We use $I=U_{s}\cap U_{c}$, the intersection of the user lists of S and C, as a substitute for C’s user list $U_{c}$ because the scale of $U_{s}$ is so big that the number of items (user identities) in $U_{c}$ that are out of the intersection is relatively small enough to be ignored; (2) These two user lists $U_{s}$ and $U_{c}$ are both private, each of which should not be revealed to the other party; (3) The location data of users in the hands of S are private from C.

Our Contributions. We proposed two-party privacy-preserving aggregate query protocols for three types of queries—RNN Cardinality query, Average Distance query and Maximum Distance query for facility location selection. Compared with the state-of-the-art scheme [3], our protocols are one step closer to practical application in terms of efficiency. Specifically, our main contributions are as follows:

1. We propose a new construction that we call a PSI filter, which can be used by two parties to filter irrelevant data corresponding to items out of the intersection without any computations and without revealing any information of items in their private sets to each other. Specifically, we extract the PSI process of [4] and modify it to a PSI filter generation protocol. The generated filter F consists of two parts, in both of which the items are indistinguishable (looks random). These two parts are held by these two parties (S and C), respectively, and they can help to find the valid data in our aggregate query protocols.
2. We propose privacy-preserving aggregate query protocols for three types of queries—RNN Cardinality query, Average Distance query and Maximum Distance query by using our PSI filter to find the valid data (corresponding to the items in the intersection) and using a Paillier cryptosystem to achieve the processing on the ciphertext. Compared with the state-of-the-art schemes [3], the use of a PSI filter rather than a superset (for hiding user identities and serving as an index) of user identity space can bring a new advantage: no need to increase the communication costs for hiding the user identity lists from each other. Further, it can efficiently reduce the times of encryption from $\tilde{n}$ to $n_{s}$ by removing many invalid computations where $\tilde{n}\left(\gg n_{s}\right)$ is the size of the superset and $n_{s}$ is the size of LSP’s user set.
3. We propose a ciphertext matrix compressing (CMC) method that can further reduce the communication cost (and the computation cost incidentally) of the RNN Cardinality query protocol then we give the optimized version of the RNNC query protocol utilizing CMC. Although our original three types of query processing are similar to some extent, the communication cost of the RNN cardinality query protocol is $O(n_{s}\cdot k)$, k× that of the other two protocols, where *k* is the number of facilities. Our ciphertext matrix compressing method can reduce the communication cost to $O(n_{s}\cdot \left\lceil k/\eta \right\rceil )$, where η is relevant to the security parameter λ and a threshold value α (Section 6). What is striking is that our compressing method has a broader scope of application; more specifically, it has great potential in small-value plaintext scenarios for compressing the scale of ciphertext while maintaining the homomorphic property.
4. We give the security analysis and performance evaluation of the aggregate query protocols. First, we prove the security of a PSI filter generation protocol, based on which we illustrate that the aggregate query protocols are privacy-preserving for the location data owned by the LSP and user lists of both the LSP and the business. Finally, we give the theoretical analysis and experimental results of performance.

The remainder of this paper is organized as follows. After reviewing the related research contents and giving the preliminaries in Section 2, we introduce our system model and problem definitions in Section 3. In Section 4, we give the definition of a PSI filter and the generation protocol that we utilized to construct the three types of query protocols in Section 5. We present a ciphertext matrix compressing method in Section 6 to further improve the efficiency of the RNNC query protocol. In Section 7, we give the proofs of correctness and privacy. In Section 8, we give the theoretical analysis and experimental results for performance evaluation. Finally, the concluding remarks are given in Section 9.

## 2. Related Work and Preliminaries

### 2.1. Related Work

#### 2.1.1. Aggregate Query on Location Data

The nearest neighbor (NN) query has received vast attention in the spatial database research community since its introduction in [5]. We say point A is the nearest neighbor of B when A is closer to B than any other points under consideration, and naturally, B is one of the reverse nearest neighbors of A. The RNN query was firstly considered in [6]. In monochromatic RNN [7,8], all points are regarded as in the same class; in contrast, the dichromatic RNN [2,6,9,10] considers two different classes of points—objects and sites. The average distance (for $L_{1}$ distance) query was first considered in min-dist optimal location query [11]. Then, [12] gave a scheme for the $L_{2}$ distance version. Recently, for the TIPS problem (Trajectory-aware Inconvenience-minimizing Placement of Services), [13] showed both the MAX-TIPS and AVG-TIPS are NP-hard. Then, a novel query called the reverse nearest neighborhood (RNH) query was proposed in [14]. Unlike an RNN query, an RNH query emphasizes a group of users instead of an individual user.

Most of the schemes of aggregate queries on location data assume that data is public or allowed to be sent to another party. Today, when people attach great importance to data privacy, these schemes do not meet our requirements.

#### 2.1.2. Privacy-Preserving Aggregate Query

The privacy-preserving database query was first defined in [15]; Ref. [16] then gave a solution. Ref. [17] showed how to execute SQL aggregations over encrypted data. The authors developed an enhanced encrypted data storage model and introduced formal query implementation techniques to translate original aggregation queries to a form that can be directly executed over the encrypted data. Ref. [18] proposed a privacy-preserving range query protocol. Ref. [19] proposed a privacy-aware query processing framework Casper for privacy-preserving NN query. Then, ref. [20] gave efficient protocols for privacy-preserving *k*-NN searches by using secure multi-party computation technologies. Ref. [21] proposed a simple privacy-preserving protocol PDAS for computing and verifying queries in outsourced databases against malicious adversaries. For the same problem in [20], ref. [22] gave a solution using a Paillier cryptosystem. Recently, ref. [3] proposed two types of solutions for privacy-preserving aggregate query protocols. The main difference between these two types is who (the server or the client) does most of the encryptions and decryptions. No matter who does, the protocols need a superset of size $\tilde{n}$ in order to conceal the identities of users and to serve as an index—the positions of any users are fixed where both the two parties can operate directly on the corresponding positions.

How to cancel the use of a superset and filtrate data without an “index” for aggregate queries on location data while the two parties both have their own private user sets is not solved yet. In general, we use homomorphic encryption and the idea of PSI to design our protocols. Compared with the traditional PSI protocols, our newly constructed PSI filter generation protocol does not find the intersection directly due to privacy issues. What the two parties get are the sets containing computationally indistinguishable labels that were generated in the protocol. In comparison with [3], we cancel the use of a superset through the use of our new primitive PSI filter and further improve the efficiency by compressing multiple data into one block of ciphertext.

### 2.2. Preliminaries

#### 2.2.1. Private Set Intersection

A private set intersection (PSI) protocol enables two parties, the Sender and the Receiver, holding private sets *X* and *Y*, respectively, to jointly compute the intersection X∩Y without revealing any additional information about their respective sets (the conditions may be weakened or changed in different circumstances). In a one-way version of PSI, only the Receiver can learn the intersection X∩Y, while the Sender learns nothing. In a concrete setting called labeled PSI [23] for applications, there is a label $l_{i}$ for each item $x_{i}\in X$, and the protocol enables the Receiver to learn the labels corresponding to the items in the intersection, i.e., the Receiver should learn the set $\left\{\left(x_{i},l_{i}\right):x_{i}\in Y\right\}$. A labeled PSI can be seen as a variant of one-way PSI.

We construct our protocols using a PSI filter (Section 4), which is generated by a modified one-way PSI. The generation process is particularly similar to a labeled PSI protocol, and the difference is that the labels do not exist before our PSI processing. In other words, the labels corresponding to the items will be generated in the intermediate process. Moreover, the Receiver does not know the specific correspondence of any pair $\left(x_{i},l_{i}\right)$. Note that for reducing the communication/computation cost, the (generated) corresponding labels are important for filtrating data in queries. For consistency, we will use the titles *Server* (S)/*Client* (C) instead of *Sender*/*Receiver*, and accordingly, use $U_{s}/U_{c}$ instead of X/Y, afterward.

#### 2.2.2. Decisional Diffie–Hellman Assumption

The decisional Diffie–Hellman (DDH) assumption is a computational hardness assumption in cyclic groups. It indicates that no efficient algorithm can distinguish between these two distributions $\left(g,g^{a},g^{b},g^{ab}\right)$ and $\left(g,g^{a},g^{b},g^{c}\right)$, which enables one to construct efficient cryptographic systems with strong security properties.

Formally, let G(λ) be a cyclic group of order *q* with secure parameter λ. For a probabilistic adversary (algorithm) A, we define the advantage of A as

(1) $$\begin{array}{cc} \mathrm{Adv}[A,G]\;= & |\mathrm{Pr}\left[A\left(g,g^{a},g^{b},g^{ab}\right)=1\right]-\mathrm{Pr}\left[A\left(g,g^{a},g^{b},g^{c}\right)=1\right]| \end{array}$$

where the probability is over a random generator *g* of G, and random a,b,c∈G. We say that the decisional Diffie–Hellman assumption holds in group G if for any probabilistic adversary A, the advantage Adv[A,G] is negligible.

#### 2.2.3. Homomorphic Encryption

Homomorphic encryption is a cryptographic primitive that allows computation on ciphertexts without decryption. The homomorphism can be expressed as $\mathrm{Enc}\left(m_{1}\right)\hat{\circ }\mathrm{Enc}\left(m_{2}\right)=\mathrm{Enc}\left(m_{1}\circ m_{2}\right)$, more strictly, $\mathrm{Dec}\left(\mathrm{Enc}\left(m_{1}\right)\;\hat{\circ }\;\mathrm{Enc}\left(m_{2}\right)\right)=m_{1}\circ m_{2}$. Note that in nondeterministic encryption, the previous homomorphic equation is not directly equivalent due to the existence of random numbers. It represents that $\mathrm{Enc}\left(m_{1}\right)\;\hat{\circ }\;\mathrm{Enc}\left(m_{2}\right)$ equals one of the ciphertexts of the message $m_{1}\circ m_{2}$.

We choose a Paillier cryptosystem [24] to achieve the additive homomorphism, i.e., $\mathrm{Enc}\left(m_{1}\right)\cdot \mathrm{Enc}\left(m_{2}\right)=\mathrm{Enc}\left(m_{1}+m_{2}\right)$. After selecting the public key pk=(n,g) and the private key sk=(λ,μ), for message m∈M, the encryption algorithm computes ciphertext $c=g^{m}\cdot r^{n}\;\mathrm{mod}\;n^{2}$ where *r* is randomly selected from $Z_{n}^{*}$. The decryption algorithm recovers the message by computing $m=L(c^{\lambda }\;\mathrm{mod}\;n^{2})\cdot \mu \;\mathrm{mod}\;n$ where $L\left(x\right)=\frac{x-1}{n}$. The Paillier cryptosystem provides semantic security against chosen-plaintext attacks (IND-CPA) under the decisional composite residuosity assumption (DCRA). In other words, the encryptions of different messages are computationally indistinguishable without knowledge of the private key.

#### 2.2.4. Security Definition

We consider two-party privacy-preserving aggregate query protocols in a semi-honest model, i.e., the adversaries are honest but curious, where the main security issue is to protect the privacy of the two parties. We formally define the security of a two-party protocol by using a simulation paradigm [25,26], which gives us an effective method to prove the security.

Let $f=(f_{1},f_{2})$ be a probabilistic polynomial-time functionality and let π be a two-party protocol for computing *f*. The view of the *i*th party (i∈{1,2}) during the execution of π on (x,y) and security parameter *n* is denoted by ${\mathrm{view}}_{i}^{\pi }\left(x,y,n\right)$ and equals $\left(w,r^{i};m_{1}^{i},\ldots ,m_{t}^{i}\right)$, where w∈{x,y} (depends on the value of *i*), $r^{i}$ equals the contents of the *i*th party’s internal random tape and $m_{j}^{i}$ represents the *j*th message that it received. The output of the *i*th party during an execution of π on (x,y) and security parameter λ is denoted by ${\mathrm{output}}_{i}^{\pi }\left(x,y,\lambda \right)$ and can be computed from its own view of the execution. We denote the joint output of both parties by ${\mathrm{output}}^{\pi }\left(x,y,\lambda \right)=\left({\mathrm{output}}_{1}^{\pi }\left(x,y,\lambda \right),{\mathrm{output}}_{2}^{\pi }\left(x,y,\lambda \right)\right)$.

**Definition** **1.** *
(Security for Semi-honest Adversaries)* *Let* $f=(f_{1},f_{2})$ *be a functionality. We say that π securely computes f in the presence of static semi-honest adversaries if there exist probabilistic polynomial-time algorithms* ${\mathrm{SIM}}_{1}$ *and* ${\mathrm{SIM}}_{2}$ *such that*

$$\begin{array}{cc} \; & \left\{{\mathrm{SIM}}_{1}\left(1^{\lambda },x,f_{1}\left(x,y\right)\right),f\left(x,y\right)\right){\}}_{x,y,\lambda }\\ & \overset{c}{\equiv }\left\{{\mathrm{view}}_{1}^{\pi }\left(x,y,\lambda \right),{\mathrm{output}}^{\pi }\left(x,y,\lambda \right)\right){\}}_{x,y,\lambda },\;\mathrm{and}\\ \; & \left\{{\mathrm{SIM}}_{2}\left(1^{\lambda },x,f_{2}\left(x,y\right)\right),f\left(x,y\right)\right){\}}_{x,y,\lambda }\\ & \overset{c}{\equiv }\left\{{\mathrm{view}}_{2}^{\pi }\left(x,y,\lambda \right),{\mathrm{output}}^{\pi }\left(x,y,\lambda \right)\right){\}}_{x,y,\lambda }, \end{array}$$

*where* $x,y\in {\left\{0,1\right\}}^{*}$ *such that* |x|=|y|*, and* n∈N.

The simulation paradigm is one of the most important paradigms in the definition and design of cryptographic primitives. In a two-party setting, it can ensure one has not learned anything about the other’s secret by showing that the one could have simulated the entire interaction by itself. In other words, one can gain no further knowledge as the result of interacting with the other beyond what it could have discovered by itself. It gives a formal way to prove the security; the details of the proof are shown in Section 7.

## 3. Models and Problem Definitions

### 3.1. System Model

**Figure 1:**
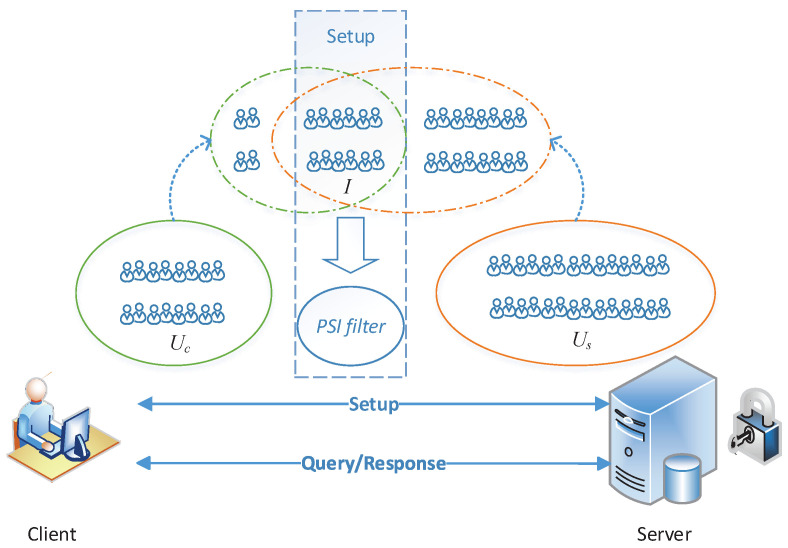
System Model.

The system model is composed of two parties, as shown in Figure 1: the server S provides location-based services and the client C that is interested in some aggregated data (query results) that are helpful for selecting the optimal facility location.

S has a private user set $U_{s}=\left\{v_{1},\ldots ,v_{n_{s}}\right\}$, where $u_{i}$ is the unique ID of the *i*th user of S. Further, S has the knowledge of all the users’ locations $L=\{l_{1},\ldots ,l_{n_{s}}\}$, where $l_{i}$ is the location of $u_{i}$. (S’s own data privacy—how it prevents getting compromised—is not in our consideration. Some techniques can be found in related fields, such as hardware security and database security. We focus on the privacy-preserving interactive protocols between S and C.) Actually, $l_{i}$ can be the expression of a point with respect to any coordinate system, for example, $l_{i}=\left(x_{i},y_{i}\right)$ in Cartesian coordinate system and $l_{i}=\left(lng_{i},lat_{i}\right)$ in geographic coordinate system. C has a private user set $U_{c}=\left\{u_{1},\ldots ,u_{n_{c}}\right\}$, where $v_{i}$ is the unique ID of the *i*th user of C. In addition, C has $F=\{f_{1},\ldots ,f_{k}\}$, a list of locations of (k−1) existing facilities and one of the candidate locations, which can be public to others.

As described in Figure 1, there are two phases of interactions: the setup phase and the query phase. In the setup phase, C and S interact to do some pre-computations where a PSI filter will be generated on the basis of the two user sets $U_{c}$ and $U_{s}$, in order to facilitate the query phase. After finishing the setup phase, when C wants to do a query, it can send a query request with a specific query type to S. After receiving the query request, S interacts with C to jointly compute an aggregate query result that will be obtained by C while protecting the privacy of both C and S. We discuss the details of privacy requirements and list all involving sensitive information in Section 3.2 There is a good property that a fixed C collaborating with S can process an aggregate query many times with just one setup phase, so the computation and communication costs of the setup phase can be amortized over a number of queries.

### 3.2. Threat Model

Both C and S are considered as semi-honest (also called honest-but-curious) adversaries [27], as described in Section 2.2.4 That is, they honestly act according to their prescribed actions in the designed protocols but may try to learn additional information from the encrypted data and all the intermediate messages obtained by themselves, and there is evidently no collusion between C and S. We say a two-party protocol π is secure in the semi-honest model if no party of the two gains information about the other’s private inputs, other than what can be deduced from the result of the protocol.

In our system model, there is some private information that needs to be hidden as follows.

- $U_{s}$: these user identities should not be revealed to C;
- $U_{c}$: these user identities should not be revealed to S;
- *L*: the user location data is private to C;
- *Q*: the query result is private to S.

Note that the values of $n_{s}$, $n_{c}$ and $n_{I}$ are actually not sensitive information. We do not hide them in our protocol, but just in case, we give a simple way to hide $n_{s}$ and $n_{c}$ to some degree. Moreover, the location that was finally selected is not private information, while the query result *Q* is private to S because the final location is visible to anyone.

### 3.3. Problem Definitions

Different sorts of aggregate queries can employ the PSI filter proposed in Section 4 to build privacy-preserving query protocols. In our system model, a privacy-preserving aggregate query protocol is a secure two-party computation protocol for functionality $Q:\left(\left(U_{c},F\right),\left(U_{s},L\right)\right)\to \left(Q,⊥\right)$, where $\left(U_{c},F\right)$ is C’s input and $\left(U_{s},L\right)$ is S’s input. After executing the protocol, C receives the query result *Q* corresponding to a specific query type while S gains nothing. Note that it is a sketchy representation of the functionality Q ignoring some inputs such as secure parameters and keys, and the input of functionality Q has not changed from the previous state-of-the-art scheme. For popularity (usefulness and usage frequency) and ease of comparison with the previous literature, we consider three types of queries, the same as in [3]—RNN Cardinality (RNNC) query, Average Distance (AVGD) query, and Maximum Distance (MAXD) query. The formal definitions of the three types of queries are given as follows:

**Definition** **2.** *
(RNNC Query)* *Given a set of locations of facilities*$F=\{f_{1},\ldots ,f_{k}\}$*, RNNC query intents to find* $\mathrm{RNNC}\left(F\right)=\{q_{i}=|\left\{u_{j}|f_{i}isNNofu_{j}\right\}{\left|\right\}}_{i\in [k]}$ *where* |·| *means the cardinality of a set. In other words, it intents to find the cardinality of RNN for each facility *$f_{i}$.

For the RNNC query, the query result Q=RNNC(F) is a *k*-vector (ordered set) of RNN cardinalities.

**Definition** **3.** *
(AVGD Query)* *Given a set of locations of facilities* $F=\{f_{1},\ldots ,f_{k}\}$*, AVGD query intends to find*

(2) $$\mathrm{AVGD}\left(F\right)=\frac{\sum_{i=1}^{n_{c}}d(u_{i},f_{j})}{n_{c}}$$

*where* $f_{j}$ *is the NN of* $u_{i}$*, and* $d(u_{i},f_{j})$* means the distance between* $u_{i}$ and $f_{j}$*. In other words, it intends to find the average distance of all users and their nearest facilities.*

**Definition** **4.** *
(MAXD Query)* *Given a set of locations of facilities* $F=\{f_{1},\ldots ,f_{k}\}$*, MAXD query intends to find* $\mathrm{MAXD}\left(F\right)=\max(d\left(u_{i},f_{j}\right))$*, where* $f_{j}$ *is the NN of* $u_{i}$*. In other words, it intends to find the maximum distance between a user* $u_{i}\in I$ *and its nearest facility* $f_{j}\in F$.

Note that we consider Manhattan distance ($L_{1}$ distance) instead of Euclidean distance ($L_{2}$ distance), because $L_{1}$ distance is more accurate for representing the driving distance in a city road network [28].

### 3.4. Design Objectives

To meet the anticipated requirements under the aforementioned system model and the threat model, the proposed protocols aim to simultaneously achieve the following objectives:

- Correctness. The client should correctly obtain the desired query result if the two parties execute the protocol honestly.
- Privacy. The private information $U_{s}$, $U_{c}$, *L* and *Q* should be hidden throughout the whole protocol. Although we use *I* as a substitute for $U_{c}$, the client does not gain any knowledge of $U_{s}$; in other words, the client has no idea if an item in $U_{c}$ also exactly belongs to $U_{s}$, and similarly, the server gains nothing about $U_{c}$.
- Efficiency. Our protocol should be efficient for both S and C. That is, both the computation cost and the communication cost should be low to support the aggregate queries with large-scale location datasets and user lists.

## 4. PSI Filter

**Definition** **5.** *(PSI filter) For a two-party query protocol π, and two datasets*$D_{1}$*and*$D_{2}$*held by*S*and*C*, respectively, let*$F=(\Theta_{1},\Theta_{2})$*be a tuple of two formally analogous sets that are held by*S*and*C*, respectively, where*$\Theta_{1}=\left\{label_{1}^{S},\ldots ,label_{|D_{1}|}^{S}\right\}$, $\Theta_{2}=\left\{label_{1}^{C},\ldots ,label_{|D_{1}⋂D_{2}|}^{C}\right\}$. F*is said to be aPSI filterif it does not contain any sensitive information and the protocol π can utilize *F*to filtrate out irrelevant data corresponding to items out of the intersection*$D_{1}⋂D_{2}$*with no extra computation.*

Our newly constructed PSI filter is a pair of special sets of labels designed to filtrate data, which means helping C to find and retain the relevant data corresponding to the items (users) in the intersection and discard other irrelevant data without revealing the private identities of users to either C or S. As described in Section 2.2 the process of generating the PSI filter is, in fact, a modified one-way PSI that is similar to a labeled PSI. Concretely, C and S hold private sets $U_{c}={\left\{u_{i}\right\}}_{i\in [n_{c}]}$, $U_{s}={\left\{v_{i}\right\}}_{i\in [n_{s}]}$, respectively. An originally non-existent label $label_{i}$ that looks random will be generated for each item $v_{i}\in U_{s}$ in the intermediate process, and C will learn $\left\{label_{i}:v_{i}\in U_{c}\right\}$, the set of labels corresponding to the items in the intersection as a result. It should be noted that in the PSI filter generating process, C does not know the specific correspondence of any pair $\left(v_{i},label_{i}\right)$, and S does not know which labels C will retain, so the identities are hidden for both C and S.

**Figure 2:**
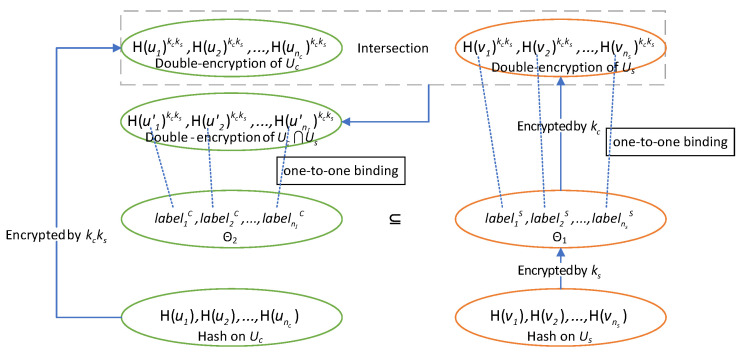
The Idea of Generating the PSI Filter.

**Figure 3:**
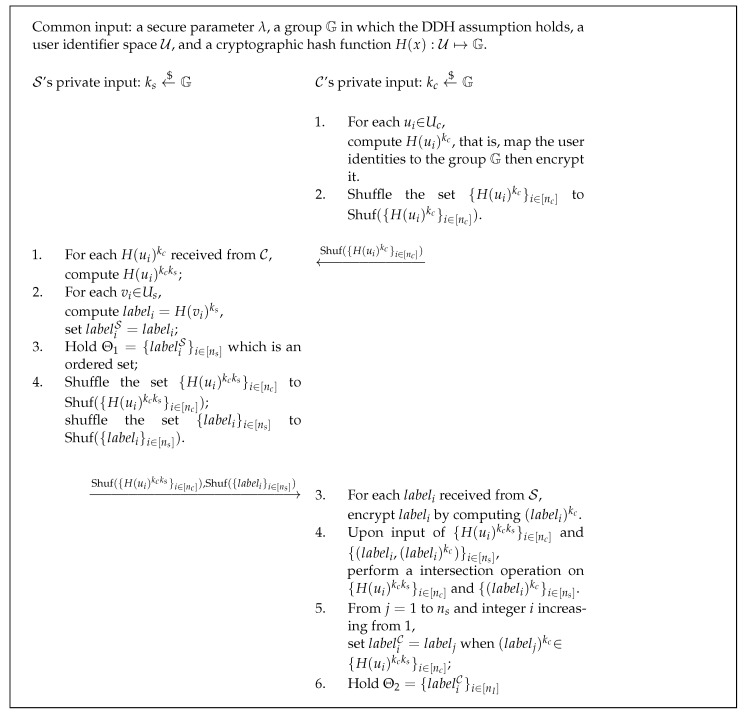
The *PSI Filter* Generation Protocol PFGen.

We give the basic idea of the PSI filter generation protocol design in Figure 2. C and S hold their private keys $k_{c}$ and $k_{s}$, respectively. For each item, $v_{i}\in U_{s}$, S computes $H{\left(v_{i}\right)}^{k_{s}}$ as the distinguishable labels forming the set $\Theta_{1}$ and specifying all the correspondence $<v_{i},H{\left(v_{i}\right)}^{k_{s}}>$. To determine if $u_{i}=v_{j}$, C and S jointly compute the double-encryption results $H{\left(u_{i}\right)}^{k_{c}k_{s}}$ and $H{\left(v_{j}\right)}^{k_{c}k_{s}}$, which is similar to Diffie–Hellman key exchange process. We have that if $u_{i}=v_{j}$ then $H{\left(u_{i}\right)}^{k_{c}k_{s}}=H{\left(v_{j}\right)}^{k_{c}k_{s}}$, and the converse proposition holds with an overwhelming probability close to 1 while the collision resistance holds. After the intersection operation on the sets ${\left\{H{\left(u_{i}\right)}^{k_{c}k_{s}}\right\}}_{i\in [n_{c}]}$ and ${\left\{H{\left(v_{j}\right)}^{k_{c}k_{s}}\right\}}_{j\in [n_{s}]}$, while guaranteeing the privacy of identities $u_{i}$ and $v_{j}$ due to the DDH assumption, C could get the subset $\Theta_{2}$ of the distinguishable labels in which the labels are corresponding to the items in $U_{c}$ one by one. The details of the PSI filter generation protocol PFGen are given in Figure 3.

Thus far, the PSI filter $F=(\Theta_{1},\Theta_{2})$ has been generated and $\Theta_{1},\Theta_{2}$ are held separately by S and C. Note that $\Theta_{1}$ is required to be an ordered set while $\Theta_{2}$ is an ordinary set. The generation protocol PFGen will be used as a subprotocol in the setup phase of aggregate query protocols. We will formally state and prove the security of the PFGen protocol in Section 7, and we just simply indicate here that the PFGen protocol is secure against semi-honest adversaries, so it can be used to construct privacy-preserving query protocols. Note that the difference from a traditional PSI protocol is that both the two parties have no idea if an item is exactly in the intersection *I*. All they can get are the labels that look random.

## 5. Aggregate Query Protocols

In this section, we use the PFGen protocol proposed in Section 4 to build privacy-preserving query protocols for different types of aggregate queries. In these protocols, the client C and the server S execute the PFGen protocol to generate a PSI filter in the setup phase. In the query phase, C finds out the encrypted data related to the users in the intersection from all candidate structured data received from S by comparing the labels in $\Theta_{1}$ with those in $\Theta_{2}$.

### 5.1. RNNC Query

Setup:

**Step 1:**S and C execute the PFGen protocol with secure parameter λ. The output is the PSI filter$F=(\Theta_{1},\Theta_{2})$, where $\Theta_{1}$ and $\Theta_{2}$ are held by S and C, respectively.

**Step 2:**S takes the same secure parameter λ to generate the key pair (sk,pk) of the Paillier cryptosystem, then sends pk to C.

Query:

**Step 1:**C sends the query request $F=\{f_{1},\ldots ,f_{k}\}$ with the type of query “RNNC” to S.

**Step 2:** After receiving the facility locations, S computes $d\langle f_{i},l_{j}\rangle$ for i∈[k] and $j\in [n_{s}]$, which means the distances between $f_{i}\in F$ and $u_{j}\in U_{s}$. S determines each user’s nearest neighbor.

**Step 3:**S initializes a $n_{s}\times k$ zero matrix M, then sets $m_{ij}=1$ for all *i*, *j* satisfying $f_{j}$ is $u_{i}$’s nearest neighbor. S computes $M^{\prime }$, the ciphertext of M under the public key pk, i.e., computes each $m_{ij}^{\prime }=\mathrm{Enc}\left(pk,m_{ij}\right)$.

**Step 4:**S augments the ciphertext matrix $M^{\prime }$ with $\Theta_{1}$, and gets $n_{s}\times \left(k+1\right)$ matrix $N^{\prime }=\begin{array}{cc} \Theta_{1} & M^{\prime } \end{array}$ as a result (treating the ordered set $\Theta_{1}$ as a column vector, the same below), then sends $N^{\prime }$ to C.

**Step 5:** For each row of $N^{\prime }$, C retains the row if the first element of the row is also in $\Theta_{2}$. Otherwise, it discards the row. Then, C discards the first column to obtain a $n_{I}\times k$ matrix $Y^{\prime }$.

**Step 6:**C multiplies the elements in each column of $Y^{\prime }$ and gets the vector $s^{\prime }=\left[s_{1}^{\prime },\ldots ,s_{k}^{\prime }\right]$. C picks a random vector $r=[r_{1},\ldots ,r_{k}]$ where $r_{i}\in Z_{n}$, computes $r^{\prime }=\left[r_{1}^{\prime },\ldots ,r_{k}^{\prime }\right]$ where $r_{i}^{\prime }=\mathrm{Enc}\left(r_{i}\right)$, then computes $t^{\prime }=\left[t_{1}^{\prime },\ldots ,t_{k}^{\prime }\right]$ where $t_{i}^{\prime }=s_{i}^{\prime }r_{i}^{\prime }$. C sends $t^{\prime }$ to S.

**Step 7:**S decrypts $t^{\prime }$ by computing $t_{i}=\mathrm{Dec}\left(sk,t_{i}^{\prime }\right)$, then returns $t=[t_{i},\ldots ,t_{k}]$ to C.

**Step 8:**C computes s=t−r and lets $q_{i}=s_{i}$ to obtain $Q=[q_{1},\ldots ,q_{k}]$ as the query result.

The key parts are (1) C/S hides its user set from the other one by using the PSI filter, which also reduces the computational cost and communication cost, (2) S hides the user’s location information by using additively homomorphic encryption, (3) C picks a random vector r to mask s (on ciphertext) to hide the query result from S. Clearly, $Q=[q_{1},\ldots ,q_{k}]$ is the desired query result where $q_{i}$ is equal to the RNN cardinality of $f_{i}$. We will give the other two aggregate query protocols with similar ideas, and a detailed explanation of correctness and privacy are given in Section 7.

In practice, it is not necessary to generate a key pair of Paillier cryptosystems every time a client comes in. The server can set several key pairs $\left\{sk_{i},pk_{i}\right\}$ corresponding to different security levels (parameters $\lambda_{i}$) and make all $\left\{\lambda_{i},pk_{i}\right\}$ public. When a new client comes in and needs to set up the server, it can pick an appropriate public key and just tell the server which one it chose. The same goes for the next two query protocols in Section 5.2 and Section 5.3

### 5.2. AVGD Query

Setup:

The setup phase is the same as in Section 5.1.

Query:

**Step 1:**C sends the query request $F=\{f_{1},\ldots ,f_{k}\}$ with the type of query “AVGD” to S.

**Step 2:** After receiving the facility locations, S computes $d\langle f_{i},l_{j}\rangle$ for i∈[k] and $j\in [n_{s}]$, which means the distances between $f_{i}\in F$ and $u_{j}\in U_{s}$. S determines each user’s nearest neighbor.

**Step 3:**S initializes a column vector $D={\left[d_{1},\ldots ,d_{n_{s}}\right]}^{T}$ where $d_{i}$ is the distance between $u_{i}$ and its nearest neighbor, then computes $D^{\prime }$, the ciphertext of D under the public key pk, i.e., computes each $d_{i}^{\prime }=\mathrm{Enc}\left(pk,d_{i}\right)$.

**Step 4:**S augments ciphertext matrix $D^{\prime }$ with $\Theta_{1}$, gets $n_{s}\times 2$ matrix $N^{\prime }=\begin{array}{cc} \Theta_{1} & D^{\prime } \end{array}$, then sends $N^{\prime }$ to C.

**Step 5:** For each row of $N^{\prime }$, C retains the row if the first element of the row is also in $\Theta_{2}$. otherwise, it discards the row. Then, C discards the first column to obtain a $n_{I}$-dimensional column vector $Y^{\prime }={\left[y_{1}^{\prime },\ldots ,y_{n_{I}}^{\prime }\right]}^{T}$ and saves $n_{I}$.

**Step 6:**C picks a random number $r\in Z_{n}$ and lets $r^{\prime }=\mathrm{Enc}\left(r\right)$. C computes $s^{\prime }=\prod_{i=1}^{n_{I}}y_{i}^{\prime }$ and $t^{\prime }=s^{\prime }r^{\prime }$, then sends $t^{\prime }$ to S.

**Step 7:**S decrypts $t^{\prime }$ by computing $t=\mathrm{Dec}(sk,t^{\prime })$, then returns *t* to C.

**Step 8:**C computes s=t−r and $Q=s/n_{I}$ to obtain the query result *Q*.

### 5.3. *MAXD* Query

Setup:

The setup phase is the same as in Section 5.1.

Query:

**Step 1:**C sends the query request $F=\{f_{1},\ldots ,f_{k}\}$ with the type of query “MAXD” to S.

**Step 2:** After receiving the facility locations, S computes $d\langle f_{i},l_{j}\rangle$ for i∈[k] and $j\in [n_{s}]$, which means the distances between $f_{i}\in F$ and $u_{j}\in U_{s}$. S determines each user’s nearest neighbor.

**Step 3:**S initializes a column vector $D={\left[d_{1},\ldots ,d_{n_{s}}\right]}^{T}$ where $d_{i}$ is the distance between $u_{i}$ and its nearest neighbor, sorts all $d_{i}\in D$ to obtain $\hat{D}={\left[d_{\tau (1)},\ldots ,d_{\tau (n_{s})}\right]}^{T}$ where $d_{\tau (i)}\geq d_{\tau (i+1)}$, then sets ${\hat{\Theta }}_{1}={\left[label_{\tau (1)}^{S},\ldots ,label_{\tau (n_{s})}^{S}\right]}^{T}$.

**Step 4:**S computes ${\hat{D}}^{\prime }={\left[d_{\tau (1)}^{\prime },\ldots ,d_{\tau (n_{s})}^{\prime }\right]}^{T}$ where $d_{\tau (i)}^{\prime }=\mathrm{Enc}\left(pk,d_{\tau (i)}\right)$, then sends $N^{\prime }=\begin{array}{cc} {\hat{\Theta }}_{1} & {\hat{D}}^{\prime } \end{array}$ to C.

**Step 5:** From the top to bottom of $N^{\prime }$, C finds out the first row in which the $label_{\tau (j)}^{S}$ is in $\Theta_{2}$ (suppose it is the *j*th row). C holds $d_{\tau (j)}^{\prime }$ and discards all other elements.

**Step 6:**C picks a random number $r\in Z_{n}$, computes $r^{\prime }=\mathrm{Enc}\left(r\right)$, then computes $t^{\prime }=d_{\tau (j)}^{\prime }r^{\prime }$. C sends $t^{\prime }$ to S.

**Step 7:**S decrypts $t^{\prime }$ by computing $t=\mathrm{Dec}(sk,t^{\prime })$, then returns *t* to C.

**Step 8:**C computes Q=t−r (in fact $Q=d_{\tau (j)}$) as the query result *Q*.

### 5.4. Application Examples

In order to make it more clear how to apply our privacy-preserving aggregate query protocols to the actual scenes, we give the application examples for each of the three types of queries as follows.

- A bank wants to open a branch.
  The bank may hope that the new branch serves more people, so it can carry out the RNNC query by interacting with the LSP. Then the bank can choose the best one from several alternative locations to open a branch.
- An express (delivery) company wants to build a new distribution station.
  The company probably hopes to minimize the total delivery distance (count the distance of all distribution stations), so it can carry out the AVGD query by interacting with the LSP. Then the company can choose the best one from several alternative locations to build a new distribution station.
- Choosing a site for a non-profit hospital to be established.
  Choosing a site for a hospital may consider the worst case—it hopes that the furthest one of the all potential patients can arrive at the hospital in a shorter time, i.e., the goal is to minimize the maximum distance between all potential patients and his/her nearest hospital, so it can carry out the MAXD query by interacting with the LSP. Then the hospital can be established in the best location chosen from several alternative locations.

## 6. Ciphertext Matrix Compressing

Although the processes of the three query protocols in Section 5 have similar design ideas, the communication costs are not exactly of the same magnitude in different query types. Concretely, the communication costs of AVGD query and MAXD query are both roughly $O(n_{s})$, but that of RNNC query is roughly $O(n_{s}\cdot k)$. The essential cause is that the dimension of matrix M initialized in Step 3 of the RNNC query must be sufficient to contain the relationship information between all $n_{s}$ users and all *k* facilities when each row of the $n_{s}$-dimension vector D in Step 3 of AVGD/MAXD query corresponds to only one (the nearest) facility. When the scale of the facility set *F* is large, the linear relationship between communication cost and the number of facilities will be a challenge. To address this issue, we compress the ciphertext for this particular scenario (the plaintext space actually used is much less than $Z_{n}$). Properly speaking, under the same security parameter, we make the same size ciphertext contain more plaintext information, which will lead to the reduction of the ciphertext matrix dimension.

In homomorphic cryptosystems satisfying CPA-security, the size of the ciphertext is bigger than that of plaintext because there must be enough randomization to achieve a “non-determination” property. In a Paillier cryptosystem, the encryption function is essentially a map $f:Z_{n}\times Z_{n}^{*}\mapsto Z_{n^{2}}^{*}$, where $Z_{n}$ is the plaintext space, $Z_{n^{2}}^{*}$ is the ciphertext space, and the selected random number $r\in Z_{n}^{*}$ ensures the “non-determination” property. Clearly, the size of the ciphertext is equal to 2× the size of the plaintext. It seems unrealistic to directly compress the ciphertext space under the premise of ensuring security: (1) It is impossible to compress $Z_{n^{2}}^{*}$ while keeping $Z_{n}$ and $Z_{n}^{*}$ fixed because *f* is bijective, (2) Ensuring a constant security level means that $Z_{n}^{*}$ can not be compressed, (3) It is hard to compress both $Z_{n}$ and $Z_{n^{2}}^{*}$ simultaneously because such behavior will easily break the ingenious bijection structure—the underlying theoretical basis of the Paillier cryptosystem. The generalized Paillier cryptosystem [29] can efficiently reduce the ciphertext expansion factor to $\frac{s+1}{s}$ where *s* is proportional to the block size of the ciphertext, but under the same secure parameter λ, picking a large *s* means concurrently expanding both plaintext space and ciphertext space. Concisely, in a generalized Paillier cryptosystem, a smaller ciphertext expansion factor leads to a larger ciphertext block size, which is not suitable for our scenario. A method [30] similar to ours is an efficient algorithm for fast batch summation using homomorphism on generalized Paillier cryptosystems, but we do not want to use the big ciphertext block because the number of facilities *k* may be relatively too small to waste a lot of space and bring unnecessary computational overhead.

We observe that even encrypting a small number needs a complete block in a Paillier cryptosystem. For a small number m≪n, the plaintext space $Z_{n}$ is far from being efficiently used, i.e., $\log_{2}\frac{n}{m}$ bits are wasted. In the RNNC query protocol, the elements in matrix $M^{\prime }$ are either Enc(0) or Enc(1); even in steps 6 and 8 for each $s_{i}^{\prime }=\mathrm{Enc}\left(s_{i}\right)$, $s_{i}$ is relatively very small compared with *n*.

**Figure 4:**
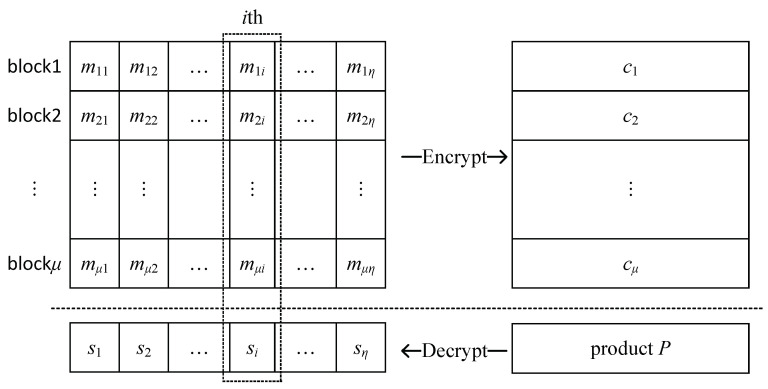
An Illustration of Compressing the Ciphertext Matrix while Keeping the Homomorphic Property Valid.

Our solution is putting multiple data (small numbers) in one plaintext while keeping the ciphertext able to be calculated using the homomorphism property from the vertical direction. Supporting the plaintext space is $Z_{n}$ and each single data in plaintext throughout the process (considering the intermediate calculation products after decrypting) is no more than α bits, i.e., no overflows of plaintext occur after the sum operations by the homomorphic property in the intermediate process. The process of ciphertext matrix compressing is given in Figure 4.

Suppose we have already generated the parameters of the Paillier cryptosystem over the security parameter λ. First, we choose the parameter α such that no overflows occur for plaintext throughout the whole query protocol and naturally let $\eta =\frac{\log_{2}n}{\alpha }$, where η means that any block${}_{i}$ can contain up to η data ($m_{i1},\ldots ,m_{i\eta }$).

Then, we perform the following process.

(1) For 1≤i≤μ, put η data in one plaintext block: for 1≤j≤η, pad each $m_{ij}=1$ or 0 to α bits with “0” then attach them end to end—a plaintext block has been constructed.

(2) For 1≤i≤μ, compute $c_{i}=\mathrm{Enc}\left(pk,{\mathrm{block}}_{i}\right)$.

(3) Compute $P=\prod_{i=1}^{\mu }c_{i}$.

(4) Compute $\hat{s}=\mathrm{Dec}\left(sk,P\right)$, then donate $\hat{s}$ as $s_{1}\left|\right|s_{2}\left||\ldots |\right|s_{\eta }$ where $s_{i}$ is a α-bit binary string.

Thus far, we have encrypted μ·η data using just μ plaintext blocks. Meanwhile, we have gotten $s_{1},\ldots ,s_{\eta }$ by running (μ−1) rather than (μ−1)·η multiplications on the ciphertext. In order to express more clearly, we give a toy example here and interpret why the homomorphic property can still be used.

*A toy example*. let λ=1024, μ=3, α=256 and $\eta =\frac{\lambda }{\alpha }=4$. Take the matrix as input

$$M=\begin{array}{c} \left[\begin{array}{cccc} 0 & 0 & 1 & 1\\ 0 & 1 & 0 & 1\\ 1 & 1 & 1 & 1 \end{array}\right]. \end{array}$$

After padding and attaching procedures, we get three blocks as follows.

(3) $$\begin{array}{cc} {\mathrm{block}}_{1}:\begin{array}{c} 0\ldots 00||0\ldots 00||0\ldots 01||0\ldots 01 \end{array}= & 2^{1\cdot \alpha }+2^{0\cdot \alpha }\\ {\mathrm{block}}_{2}:\begin{array}{c} 0\ldots 00||0\ldots 01||0\ldots 00||0\ldots 01 \end{array}= & 2^{2\cdot \alpha }+2^{0\cdot \alpha }\\ {\mathrm{block}}_{3}:\begin{array}{c} 0\ldots 01||0\ldots 01||0\ldots 01||0\ldots 01 \end{array}= & 2^{3\cdot \alpha }+2^{2\cdot \alpha }+2^{1\cdot \alpha }+2^{0\cdot \alpha } \end{array}$$

After (2)–(4), it is easy to draw that

(4) $$\begin{array}{cc} P & =\prod_{i=1}^{3}c_{i}\\ \; & =\mathrm{Enc}(\sum_{i=1}^{3}{\mathrm{block}}_{i})\\ \; & =1\times 2^{3\cdot \alpha }+2\times 2^{2\cdot \alpha }+2\times 2^{1\cdot \alpha }+3\times 2^{0\cdot \alpha }\\ \; & =0\ldots 01||0\ldots 010||0\ldots 010||0\ldots 011. \end{array}$$

Therefore, we get $s_{1}=1$, $s_{2}=2$, $s_{3}=2$ and $s_{4}=3$. It is easy to verify that $s_{j}=\sum_{i=1}^{3}m_{ij}$ for any j∈{1,2,3,4}.

For our column-wise summation problem of a small-value μ×η matrix, the proposed ciphertext matrix compressing combines (pads with 0 then links) multiple plaintexts in a row before encrypting. The number of ciphertext blocks can be reduced from μ×η to μ, and the number of encryption/decryption operations is reduced from μ×η to μ. At the same time, the homomorphic property is kept valid. Therefore, we can reform the RNNC query protocol by utilizing the ciphertext matrix compressing method. Here is the variation of the steps of the query process.

**An Improved Protocol RNNC-CMC:**

**Step 3:**S initializes an $n_{s}\times k$ zero matrix M, then sets $m_{ij}=1$ for all *i*, *j* satisfying $f_{j}$ is $u_{i}$’s nearest neighbor. S compresses the matrix M to $n_{s}\times \left\lceil k/\eta \right\rceil$ matrix $\tilde{M}$. (When *k* is not an integer multiple of η, the way to deal with the extra space is simple: padding with “0” in Step 3.) S computes ${\tilde{M}}^{\prime }$, the ciphertext of $\tilde{M}$ under the public key pk, i.e., computes each ${\tilde{m}}_{ij}^{\prime }=\mathrm{Enc}\left(pk,{\tilde{m}}_{ij}\right)$.

**Step 4:**S augments the ciphertext matrix ${\tilde{M}}^{\prime }$ with $\Theta_{1}$, and gets $n_{s}\times \left(\left\lceil k/\eta \right\rceil +1\right)$ matrix ${\tilde{N}}^{\prime }=\begin{array}{cc} \Theta_{1} & {\tilde{M}}^{\prime } \end{array}$ as a result, then sends ${\tilde{N}}^{\prime }$ to C.

**Step 5:** For each row of $N^{\prime }$, C retains the row if the first element of the row is also in $\Theta_{2}$. Otherwise, it discards the row. Then, C discards the first column to obtain a $n_{I}\times \left\lceil k/\eta \right\rceil$ matrix ${\tilde{Y}}^{\prime }$.

**Step 6:**C multiplies the elements in each column of ${\tilde{Y}}^{\prime }$ and gets the vector ${\tilde{s}}^{\prime }=\left[{\tilde{s}}_{1}^{\prime },\ldots ,{\tilde{s}}_{\left\lceil k/\eta \right\rceil }^{\prime }\right]$. C picks a random vector $r=[r_{1},\ldots ,r_{\left\lceil k/\eta \right\rceil }]$, computes $r^{\prime }=\left[r_{1}^{\prime },\ldots ,r_{\left\lceil k/\eta \right\rceil }^{\prime }\right]$ where $r_{i}^{\prime }=\mathrm{Enc}\left(r_{i}\right)$, then computes ${\tilde{t}}^{\prime }=\left[{\tilde{t}}_{1}^{\prime },\ldots ,{\tilde{t}}_{\left\lceil k/\eta \right\rceil }^{\prime }\right]$ where ${\tilde{t}}_{i}^{\prime }={\tilde{s}}_{i}^{\prime }r_{i}^{\prime }$. C sends ${\tilde{t}}^{\prime }$ to S.

**Step 7:**S decrypts $t^{\prime }$ by computing $t_{i}=\mathrm{Dec}\left(sk,{\tilde{t}}_{i}^{\prime }\right)$, then returns $\tilde{t}=\left[{\tilde{t}}_{i},\ldots ,{\tilde{t}}_{\left\lceil k/\eta \right\rceil }\right]$ to C.

**Step 8:**C computes $\tilde{s}=\tilde{t}-\tilde{r}$, then separates the vector $\left[{\tilde{s}}_{1},\ldots ,{\tilde{s}}_{\left\lceil k/\eta \right\rceil }\right]$ to $\left[s_{1},\ldots ,s_{k}\right]$—like (4) in CMC for each ${\tilde{s}}_{i}$ (ignoring separated $s_{i}$ for i>k). Finally, C sets $q_{i}=s_{i}$ to obtain $Q=[q_{1},\ldots ,q_{k}]$ as the query result.

The changes of the reformed protocol RNNC-CMC are intuitive: (1) compressing the scale of matrix M from $n_{s}\times k$ to $n_{s}\times \left\lceil k/\eta \right\rceil$, which can efficiently reduce the communication cost; (2) one homomorphic operation (multiplication) on ciphertext by column is equivalent to η operations in the original RNNC query protocol. The details of the comparison are given in Section 8.

## 7. Security Analysis

In this section, we first derive the correctness of our aggregate protocols. Then, we consider the privacy of the PSI filter generation protocol PFGen, give an optional handling for stricter privacy protection as a supplement and finally discuss the privacy of our aggregate query protocols and two types of external attackers.

### 7.1. Correctness

We simply derive the correctness of three types of queries.

For a component $q_{i}\in Q$ in RNNC query, the following equation holds:

(5) $$\begin{array}{cc} q_{i} & =t_{i}-r_{i}=\mathrm{Dec}\left(sk,t_{i}^{\prime }\right)-r_{i}=\mathrm{Dec}\left(sk,s_{i}^{\prime }r_{i}^{\prime }\right)-r_{i}\\ \; & =\mathrm{Dec}\left(sk,\prod_{j=1}^{n_{I}}y_{ji}\cdot r_{i}\right)-r_{i}=\sum_{j=1}^{n_{I}}y_{ji}+r_{i}-r_{i}=\sum_{u_{i}\in I}m_{ij}. \end{array}$$

For the query result *Q* in AVGD query,

(6) $$\begin{array}{cc} Q & =\frac{s}{n_{I}}=\frac{t-r}{n_{I}}=\frac{\mathrm{Dec}(sk,t^{\prime })-r}{n_{I}}=\frac{\mathrm{Dec}(sk,s^{\prime }r^{\prime })-r}{n_{I}}\\ \; & =\frac{\mathrm{Dec}(sk,\prod_{i=1}^{n_{I}}y_{i}^{\prime }\cdot r^{\prime })-r}{n_{I}}=\frac{\sum_{i=1}^{n_{I}}y_{i}+r-r}{n_{I}}=\frac{\sum_{u_{i}\in I}d_{i}}{n_{I}}. \end{array}$$

For the query result *Q* in MAXD query, due to the monotonicity of $\hat{D}$, i.e., $d_{\tau (i)}\geq d_{\tau (i+1)}$, and Step 5, we only need to demonstrate that $t-r=d_{\tau (j)}$ holds as follows:

(7) $$\begin{array}{cc} t-r & =\mathrm{Dec}\left(sk,t^{\prime }\right)-r=\mathrm{Dec}\left(sk,d_{\tau (j)}^{\prime }r^{\prime }\right)-r\\ \; & =d_{\tau (j)}+r-r=d_{\tau (j)}. \end{array}$$

### 7.2. Privacy

As for the privacy of our aggregate query protocols, we first show our PSI filter generation protocol PFGen proposed in Section 4 would not reveal any information about the private data and give optional handling for stricter privacy protection (treating $n_{s}$ and $n_{c}$ as privacy). Based on this, we illustrate that the proposed aggregate query protocols are privacy-preserving.

The following lemma is given to illustrate the security of the PFGen protocol in a semi-honest model, and we give the proof in the simulation paradigm.

**Lemma** **1.** *There exist PPT simulators* ${\mathrm{SIM}}_{1}$ *and* ${\mathrm{SIM}}_{2}$ *, s.t. for all security parameters λ and inputs* ${\left\{u_{i}\right\}}_{i\in [n_{c}]}$ *,* ${\left\{v_{j}\right\}}_{j\in [n_{s}]}$ *,*

$${\mathrm{REAL}}^{c,\lambda }\left({\left\{u_{i}\right\}}_{i\in [n_{c}]},{\left\{v_{j}\right\}}_{j\in [n_{s}]}\right)\overset{c}{\equiv }{\mathrm{SIM}}_{c}\left(1^{\lambda },{\left\{u_{i}\right\}}_{i\in [n_{c}]},n_{s},n_{I}\right),$$

*and*

$${\mathrm{REAL}}^{s,\lambda }\left({\left\{u_{i}\right\}}_{i\in [n_{c}]},{\left\{v_{j}\right\}}_{j\in [n_{s}]}\right)\overset{c}{\equiv }{\mathrm{SIM}}_{s}\left(1^{\lambda },{\left\{v_{j}\right\}}_{j\in [n_{s}]},n_{c},n_{I}\right).$$

**Proof.**  We give the simulators ${\mathrm{SIM}}_{c}$ and ${\mathrm{SIM}}_{s}$ in Algorithms 1 and 2 as follows.

**Algorithm 1**${\mathrm{SIM}}_{c}$: The simulator for Client
**Input:**

$\left(1^{\lambda },{\left\{u_{i}\right\}}_{i\in [n_{c}]},n_{s},|I|\right)$


**Output:**

$view_{c}={\mathrm{SIM}}_{c}(1^{\lambda },{\left\{u_{i}\right\}}_{i\in [n_{c}]},n_{s},|I|)$

1:Generate key $k_{c}\in G$2:Honestly generate and send $\mathrm{Shuf}({\left\{H{\left(u_{i}\right)}^{k_{c}}\right\}}_{i\in [n_{c}]})$ as Client’s message in Step 2.3:Create a dummy set $D_{1}={\left\{g_{i}\right\}}_{i\in [n_{c}]}$, where each $g_{i}$ is randomly selected from G.4:Compute then send $\mathrm{Shuf}({\left\{g_{i}^{k_{c}}\right\}}_{i\in [n_{c}]})$ as the message received from Server.(regard $g_{i}$ as a dummy $H{\left(u_{i}\right)}^{k_{s}}$)5:Create a dummy set $D_{2}={\left\{h_{i}\right\}}_{i\in [n_{s}]}$, where $h_{i}=g_{i}$ for i∈[|I|] and each $h_{i}$ for $i\in [|I|+1,n_{s}]$ is randomly selected from G.6:Send $\mathrm{Shuf}({\left\{h_{i}\right\}}_{i\in [n_{s}]})$ as the message received from Server. (regard $h_{i}$ as a dummy $label_{i}=H{\left(v_{i}\right)}^{k_{s}}$)7:Honestly generate Client’s message in Step 3 using $D_{2}={\left\{h_{i}\right\}}_{i\in [n_{s}]}$.8:Honestly execute Step 4 using ${\left\{g_{i}^{k_{c}}\right\}}_{i\in [n_{c}]}$ and ${\left\{h_{i},h_{i}^{k_{c}}\right\}}_{i\in [n_{s}]}$9:Output Client’s view $view_{c}$.

**Algorithm 2**${\mathrm{SIM}}_{s}$: The simulator for Server
**Input:**

$\left(1^{\lambda },{\left\{v_{i}\right\}}_{i\in [n_{s}]},n_{c}\right)$


**Output:**

$view_{s}={\mathrm{SIM}}_{s}\left(1^{\lambda },{\left\{v_{i}\right\}}_{i\in [n_{s}]},n_{c}\right)$

1:Generate key $k_{s}\in G$2:Create a dummy set $D_{3}={\left\{g_{i}^{\prime }\right\}}_{i\in [n_{c}]}$, where each $g_{i}^{\prime }$ is randomly selected from G. Send $\mathrm{Shuf}({\left\{g_{i}^{\prime }\right\}}_{i\in [n_{c}]})$ as the message received from Client.3:Honestly generate ${\left\{{g_{i}^{\prime }}^{k_{s}}\right\}}_{i\in [n_{c}]}$ and ${\left\{H{\left(v_{i}\right)}^{k_{s}}\right\}}_{i\in [n_{s}]}$.4:Compute then send both $\mathrm{Shuf}({\left\{{g_{i}^{\prime }}^{k_{s}}\right\}}_{i\in [n_{c}]})$ and $\mathrm{Shuf}({\left\{H{\left(v_{i}\right)}^{k_{s}}\right\}}_{i\in [n_{s}]})$ as Server’s message to be sent to Client.(regard ${g^{\prime }}_{i}$ as a dummy $H{\left(u_{i}\right)}^{k_{c}}$)5:Output Server’s view $view_{s}$

The simulation process of ${\mathrm{SIM}}_{c}$ is the same as C’s Step 1–4 of the PFGen protocol as long as we regard ${\left\{g_{i}\right\}}_{i\in [n_{c}]}$ and ${\left\{h_{i}\right\}}_{i\in [n_{s}]}$ as the dummy ${\left\{H{\left(u_{i}\right)}^{k_{s}}\right\}}_{i\in [n_{c}]}$ and the dummy ${\left\{label_{i}=H{\left(v_{i}\right)}^{k_{s}}\right\}}_{i\in [n_{s}]}$. Similarly, the simulation process of ${\mathrm{SIM}}_{s}$ is the same as S’s Step 1–4 of the PFGen protocol when we regard ${\left\{g_{i}^{\prime }\right\}}_{i\in [n_{c}]}$ as the dummy ${\left\{H{\left(u_{i}\right)}^{k_{c}}\right\}}_{i\in [n_{c}]}$.

We can say that for all security parameters λ and inputs ${\left\{u_{i}\right\}}_{i\in [n_{c}]}$, ${\left\{v_{j}\right\}}_{j\in [n_{s}]}$,

$${\mathrm{REAL}}^{c,\lambda }\left({\left\{u_{i}\right\}}_{i\in [n_{c}]},{\left\{v_{j}\right\}}_{j\in [n_{s}]}\right)\overset{c}{\equiv }{\mathrm{SIM}}_{c}\left(1^{\lambda },{\left\{u_{i}\right\}}_{i\in [n_{c}]},n_{s},n_{I}\right),$$

and

$${\mathrm{REAL}}^{s,\lambda }\left({\left\{u_{i}\right\}}_{i\in [n_{c}]},{\left\{v_{j}\right\}}_{j\in [n_{s}]}\right)\overset{c}{\equiv }{\mathrm{SIM}}_{s}\left(1^{\lambda },{\left\{v_{j}\right\}}_{j\in [n_{s}]},n_{c},n_{I}\right),$$

from the DDH assumption. That is, C could not distinguish $\left({\left\{g_{i}^{k_{c}}\right\}}_{i\in [n_{c}]},{\left\{h_{i}^{k_{c}}\right\}}_{i\in [n_{s}]}\right)$ and $\left({\left\{H{\left(u_{i}\right)}^{k_{c}k_{s}}\right\}}_{i\in [n_{c}]},{\left\{label_{i}=H{\left(v_{i}\right)}^{k_{c}k_{s}}\right\}}_{i\in [n_{s}]}\right)$; and S could not distinguish ${\left\{{g_{i}^{\prime }}^{k_{s}}\right\}}_{i\in [n_{c}]}$ and ${\left\{H{\left(u_{i}\right)}^{k_{c}k_{s}}\right\}}_{i\in [n_{c}]}$.    □

From Lemma 1, we know that throughout the PFGen protocol, C can know nothing more than $n_{s}$ and $n_{I}$; S can know nothing more than $n_{c}$ and $n_{I}$. These values are, in fact, not so private for S and C. When S and C want to further enhance the privacy, i.e., to hide the cardinalities $n_{s}$ and $n_{c}$ from each other, S/C can simply add some dummy users to $U_{s}$/$U_{c}$. This can efficiently conceal $n_{s}$ and $n_{c}$ to some extent, and when a new dummy user is added, after being hashed to group G, it may collide with a real user with a probability of $\frac{1}{\left|G\right|}$. It can be said that $n_{I}$ is not a privacy issue when $n_{s}$ and $n_{c}$ have been concealed.

In the query process, all the messages C received are $N^{\prime }$ and t (or *t*) where t (or *t*) is not a private message to C; and the only message S received is $t^{\prime }$ (or $t^{\prime }$). In the RNNC query protocol, $N^{\prime }=\begin{array}{cc} \Theta_{1} & M^{\prime } \end{array}$ is a ciphertext matrix augmented with a vector of randomized labels from where C can not learn any plaintext information without the private key sk, and $t^{\prime }=\left[t_{1}^{\prime },\ldots ,t_{k}^{\prime }\right]$ is a ciphertext vector where each component $t_{i}^{\prime }=s_{i}^{\prime }r_{i}^{\prime }=\mathrm{Enc}\left(pk,q_{i}+r_{i}\right)$—even a curious S decrypts $\mathrm{Enc}(pk,q_{i}+r_{i})$ by using sk, a masked query result $q_{i}+r_{i}$ would not reveal any information about $q_{i}$. In the AVGD query protocol, $N^{\prime }=\begin{array}{cc} \Theta_{1} & D^{\prime } \end{array}$ is also ciphertext from where C can not learn anything, and $t^{\prime }=s^{\prime }r^{\prime }=\mathrm{Enc}\left(Q\cdot n_{I}+r\right)$ is a masking version of $\mathrm{Enc}(Q\cdot n_{I})$ ensuring the privacy of the query result *Q*. In the MAXD query, similarly, $N^{\prime }$ reveals nothing to C, and C computes $t^{\prime }=d_{\tau (j)}^{\prime }r^{\prime }$ to ensure the privacy of the query result $Q=d_{\tau (j)}$ to S due to $\mathrm{Dec}\left(sk,t^{\prime }\right)=d_{\tau (j)}+r$. Moreover, our ciphertext matrix compressing method does not sacrifice or reduce the security level. Because of the indistinguishability of the ciphertext in the Paillier cryptosystem, for the same security parameter λ, the RNNC-CMC query protocol is strictly as secure as the original RNNC query protocol in Section 5.

In addition, traditional protocols with similar ideas to Diffie–Hellman key exchange may suffer from two attacks—statistical attack and man-in-the-middle attack. We give the discussions on these two types of attacks as follows.

*Statistical attack*. In the PFGen protocol, each user will obtain a fixed label, and the label corresponding to each user is always the same. Intuitively, it seems that a statistical attack can get the frequency of the users during the queries. In fact, when the client and the server have jointly executed the PFGen protocol, the generated PSI filter (two sets of fixed labels) has also fixed the user sets with unknown correspondences, which means the client with a modified user set $U_{c}^{\prime }$ could not successfully execute the query protocols—even the client keeps the set $\mathrm{Shuf}({\left\{label_{i}\right\}}_{i\in [n_{s}]})$, the set $\Theta_{2}$ could not be modified appropriately based only on $U_{c}^{\prime }$ without the correspondences.

*Man-in-the-middle attack*. There may be a malicious man intercepting in the middle between S and C, sending and receiving data by using another key $k_{e}$. There is a simple way to deal with this attack: using the signature technique to ensure the authenticity of the source. Specifically, the Sender (whether S or C) signs all messages as a whole during each interaction. Even without using the signature technique, it is difficult for the attacker to obtain useful information. When the attacker has jointly performed the PFGen protocol with S, the computationally indistinguishable labels are meaningless for the attacker, and they can not obtain any private information from S during some forged queries, according to Lemma 1. When the attacker pretending to be a server has jointly performed the PFGen protocol with C, the difference from the previous is that C may obtain a wrong result (non-optimal location) from the message received from the attacker during some queries. It can be said that the attacker may consume a lot of energy and can not obtain the information they want to know. Again, to prevent “man-in-the-middle attacker returning wrong query result”, the signature technique is sufficient.

## 8. Performance Evaluation

### 8.1. Theoretical Analysis

We first analyze the computational complexity for each party in the four proposed protocols and compare them with those of the state-of-the-art schemes in [3]—the server-based protocols RNNC/S, AVGD/S and MAXD/S, and the client-based protocols RNNC/C, AVGD/C and MAXD/C, as shown in Table 1. We focus on the relatively time-consuming operations, i.e., encryption, decryption, multiplication on ciphertext and exponentiation on ciphertext, and the time-saving but repetitive operation, i.e., distance calculation. Note that we ignore the constant permutation operations in Table 1. To be more intuitive, the setup phase and query phase are considered separately.

*Setup phase.* In the setup phase of all our four query protocols, S and C jointly run the PFGen protocol where both S and C need $\left(n_{s}+n_{c}\right)$ exponentiation operations modular $n=2^{\lambda }$, which is much faster than the modular $n^{2}$. In addition, S needs two permutations and C needs one permutation, both of which can be ignored. It is a small cost compared to the client-based protocols RNNC/C, AVGD/C and MAXD/C, where the client C needs to execute $\tilde{n}$ encryptions. Although the setup phase is very simple in server-based protocols RNNC/S, AVGD/S and MAXD/S, it will bring a great amount of computation cost to C in the query phase.

*Query phase.* In the query phase, for our RNNC, AVGD and MAXD query protocols, the computation processes of S are similar—$n_{s}\cdot k$ distance calculations, $n_{s}$ encryptions and 1 decryption for both the AVGD query and MAXD query, and due to the dimension issue of matrix M, $n_{s}\cdot k$ distance calculations, $n_{s}\cdot k$ encryptions and *k* decryptions for the RNNC query. The main operations of C take place in Step 6—$n_{I}\cdot k$ multiplications on the cipher and *k* encryptions for RNNC, $n_{I}$ multiplications on the cipher and one encryption for AVGD, one multiplication on cipher and one encryption for MAXD. In addition, for AVGD, C needs to execute one division in Step 8. For our RNNC-CMC protocol, we further reduce the computational complexity after the distance calculations to 1/k times of our original RNNC protocol for both S and C.

From the analysis results, since we avoid the use of a superset with the very large cardinality $\tilde{n}$ (no “$\tilde{n}$ enc” operations in our schemes), we have completely avoided a large number of homomorphic encryption operations on both the server and client-sides and the corresponding computational complexity has been reduced. Further, we avoid a big number w>max in the MAXD query protocol, resulting in a reduction of a large number of exponentiation and encryption operations. In addition, we further reduce the parameter *k* to ⌈k/η⌉ in the improved protocol RNNC-CMC.

For communication cost, we give the final result directly as follows, which is between those of the server-based protocols and the client-based protocols in [3].

(1) RNNC: $O(n_{s}\cdot k)\times$ size of the block of cipher;

(2) AVGD: $O(\frac{3}{2}n_{s}+n_{I})\times$ size of the block of cipher;

(3) MAXD: $O(\frac{3}{2}n_{s})\times$ size of the block of cipher;

(4) RNNC-CMC: $O(n_{s}\cdot \left(\left\lceil k/\eta \right\rceil +\frac{1}{2}\right))\times$ size of the block of cipher.

In summary, the proposed aggregate query protocols are comprehensively ahead of the server-based protocols and have advantages and disadvantages in some aspects compared with the client-based protocols.

### 8.2. Experimental Results

**Figure A1:**
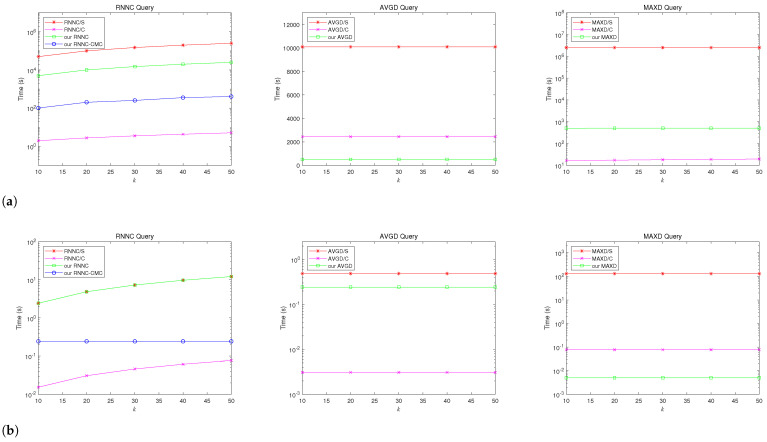
Query processing time of the protocols (vs. *k*). (**a**) Time cost of the server S. (**b**) Time cost of the client C.

**Figure A2:**
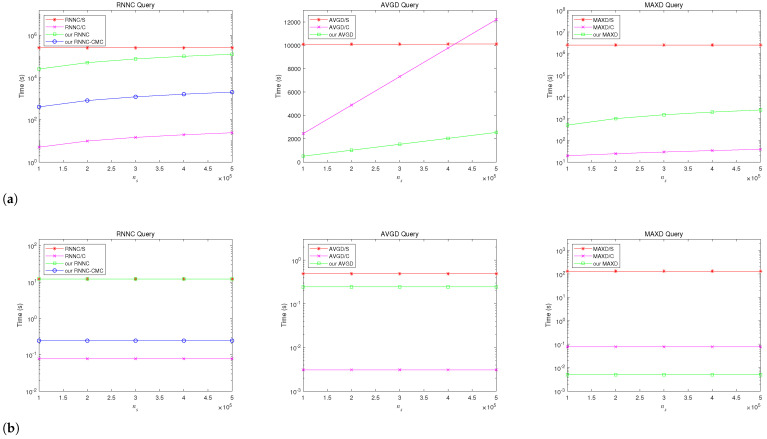
Query processing time of the protocols (vs. $n_{s}$). (**a**) Time cost of the server S. (**b**) Time cost of the client C.

We implement all our protocols in Python 3.7 with a 64-bit Windows 10 system, 3.20 GHz Intel Core i5 processor and 16 GB RAM. We choose the security parameter λ=1024, and set $n_{c}$ = 20,000 and $\tilde{n}$ = 1,000,000, the same values as in [3] for reasonable comparisons (We do not emphasize that we use the real-world dataset [31], because whether the location data are real or randomly selected has no effect on our experimental results, and without considering differential privacy, real-world datasets are meaningless for efficiency tests. In fact, the results of the experiments (the trends of the lines in Figure A1 and Figure A2) on real and unreal location datasets are exactly the same). In addition, we suppose $n_{I}=n_{c}$ in our experiment. First, for the three types of queries—RNNC, AVGD and MAXD, we fix the value $n_{s}$ = 100,000 and test the computation costs of our protocols and those of the previous along with the change of *k*. Then, we fix the value k=50 and test the computation costs along with the change of $n_{s}$. For our RNNC-CMC protocol, we set the threshold α=16, which is enough for the general RNNC query (each $q_{i}$ is no more than $2^{16}-1$ = 65,535), and accordingly, η=64.

Since the server and the client may have different scales of computing power in a real environment, we test the cost of the server and the cost of the client separately and give their lines in figures. Our experimental results are given in Figure A1 and Figure A2 in Appendix A.

There are two different types of exponentiation operations in our experiments. The first one is the exponentiation modular $n^{2}$ (2048 bits) executed on the client-side of MAXD/S and the server-side of AVGD/C and MAXD/C. The second one is the exponentiation modular *n* (1024 bits) executed in the setup phase (jointly running PFGen protocol) of our four protocols. Note that the time cost of modular exponentiation operation is very unstable when the exponent changes, and we know that computing $a^{x}$ costs the equivalent of $\log_{2}x$ multiplications by using the fast exponential algorithm. Therefore, we use the mathematical expectation instead of the unstable value. Suppose an exponent is a *b*-bit number, the expectation of equivalent times of multiplications can be calculated as $E\left(t\right)=\frac{\sum_{x=0}^{2^{b}-1}t\left(x\right)}{2^{b}}\approx \frac{\int_{1}^{2^{b}}\log_{2}xdx}{2^{b}}\approx b-1$. Therefore, we use (b−1)× the time of 1 multiplication instead of 1 exponentiation to draw the figures to clearly show the trend of change. Moreover, according to our test, the time cost of the 1024-bit modular exponentiation operation is a little smaller than a quarter of that of the 2048-bit.

From the experimental results, we give what we see for reference as follows.

(1) For the RNNC query, our protocols (RNNC and RNNC-CMC) are more efficient than RNNC/S protocols on both the client-side and the server-side, and not as efficient as the RNNC/C protocol but acceptable (Our protocols avoid a lot of encryption operations in the setup phase compared to client-based protocols (RNNC/C, AVGD/C and MAXD/C). The price of the client-based protocols being faster than ours in some cases (on the client-side for RNNC and AVGD queries, and on the server-side for RNNC and MAXD queries) is that a lot of calculations are transferred to the setup phase. In addition, taking note of the multiples of the time cost comparisons in (1), (2) and (3), $\frac{1}{80}$ and $\frac{1}{25}$ are non-ignorable values, but 0.24 s (in our experimental environment) to the client and hundreds of seconds (in our experimental environment) to the server are actually very small values). Concretely, taking k=50 and $n_{s}$ = 100,000, on the client-side, our RNNC-CMC protocol is (0.24 s) 50× faster than the RNNC/S protocol, and $\frac{1}{3}$× faster than the RNNC/C protocol; on the server-side, our RNNC-CMC protocol (407.47 s) is 620× faster than the RNNC/S protocol, and $\frac{1}{80}$× faster than the RNNC/C protocol.

(2) For the AVGD query, on the client-side, the time cost of our AVGD protocol is between that of the AVGD/S protocol and AVGD/C protocol. However, on the server-side, our protocol is much more efficient than the previous two, and it is valuable that our query protocol reduces the rate of increase in the time cost of the server when $n_{s}$ increases (Figure A2-AVGD Query). Concretely, taking k=50 and $n_{s}$ = 100,000, on the client-side, our AVGD protocol is (0.24 s) 2× faster than the AVGD/S protocol, and $\frac{1}{80}$× faster than the AVGD/C protocol; on the server-side, our AVGD protocol is (508.46 s) 20× faster than the AVGD/S protocol, and 5× faster than the AVGD/C protocol. In addition, when $n_{s}$ increases to 500,000, our AVGD protocol is (2542.30 s) 4× faster than the AVGD/S protocol, and 5× faster than the AVGD/C protocol on the server-side.

(3) For the MAXD query, our protocol is friendly to a resource-limited client compared with the two previous protocols. It is more than one magnitude faster than the client-based protocol MAXD/C. Moreover, on the server-side, the time cost of our MAXD protocol is between that of the MAXD/S protocol and MAXD/C protocol. Concretely, taking k=50 and $n_{s}$ = 100,000, on the client-side, our MAXD protocol is (0.0051 s) 26,000× faster than the MAXD/S protocol, and 15× faster than the MAXD/C protocol; on the server-side, our MAXD protocol is (508.46 s) 5000× faster than the MAXD/S protocol, and $\frac{1}{25}$× faster than the MAXD/C protocol.

## 9. Conclusions

In this paper, we have studied the aggregate queries for facility location selection between an LSP and a business with confidentiality. We proposed the construction of a PSI filter to help two parties find the relevant data corresponding to the items in the intersection without revealing the privacy of the items, including those in the intersection. Then, for location data analysis, we utilize our PSI filter to construct three types of aggregate query protocols without setting a superset of a large size for concealing the user identities and serving as an index. Moreover, we propose a ciphertext matrix-compressing method that makes one ciphertext block contain multiple small plaintext data while keeping the homomorphic property valid, and, subsequently, we give the improved protocol by utilizing this method. It further raises the efficiency of both computation and communication on the basis of the original query process. Finally, we illustrate the correctness and privacy and give the performance evaluation through theoretical analysis and experiments that show the superiorities of our protocols.

## Figures and Tables

**Table 1:** Computational Complexity.

Comp. Complexity	Setup		Query Process	
	S	C	S	C
State-of-the-art Schemes				
RNNC/S	-	-	$n_{s}\cdot k$ dist $\tilde{n}\cdot k$ enc *k* dec	$n_{c}\cdot k$ mult *k* enc
AVGD/S	-	-	$n_{s}\cdot k$ dist $2\cdot \tilde{n}$ enc 2 dec	$2\cdot n_{c}$ mult 2 enc 1 div
MAXD/S	-	-	$n_{s}\cdot k$ dist n·w enc *w* dec	$w\cdot (n_{c}-1)$ mult *w* exp
RNNC/C	-	$\tilde{n}$ enc	$n_{s}\cdot k$ dist *k* enc $n_{s}+k$ mult	*k* dec
AVGD/C	-	$\tilde{n}$ enc	$n_{s}\cdot k$ dist 2 enc $2\cdot n_{s}$ mult $n_{s}$ exp	2 dec 1 div
MAXD/C	-	$\tilde{n}$ enc	$n_{s}\cdot k$ dist $n_{s}$ mult *w* exp ≤*w* enc	w−q+1 dec
Our Schemes				
RNNC	1 PFGen		$n_{s}\cdot k$ dist $n_{s}\cdot k$ enc *k* dec	$n_{I}\cdot k$ mult *k* enc
AVGD	1 PFGen		$n_{s}\cdot k$ dist $n_{s}$ enc 1 dec	1 enc $n_{I}$ mult 1 div
MAXD	1 PFGen		$n_{s}\cdot k$ dist $n_{s}$ enc 1 dec	1 enc 1 mult
RNNC-CMC	1 PFGen		$n_{s}\cdot k$ dist $n_{s}\cdot \left\lceil k/\eta \right\rceil$ enc ⌈k/η⌉ dec	$n_{I}\cdot \left\lceil k/\eta \right\rceil$ mult ⌈k/η⌉ enc

1. The $\tilde{n}$ is the size of the superset. Typically, in order to achieve enough privacy for both $U_{s}$ and $U_{c}$, $\tilde{n}$ should be far greater than (≫) $n_{s}$, or at least $\frac{\tilde{n}}{n_{s}}$ should be not less than a specified threshold. 2. *w* is a large number satisfying w>max, where max is the maximum distance between a user and their nearest facility. 3. When S and C jointly run a PFGen processing, both S and C need $\left(n_{c}+n_{s}\right)$ exponentiation (modular *n*) operations, while the “exp” represents the exponentiation operation modular $n^{2}$. 4. The parameter $\eta =\frac{\lambda (=\log_{2}n)}{\alpha }$ is the amount of data concluded in one ciphertext block where λ is the security parameter and α is the number of bits reserved for intermediate process with no overflows.

## Data Availability

Not applicable.

## Notations

The following notations are used in this manuscript:

Notation	Description
C and S	client and server
$k_{c},k_{s}$	C,S’s secret key
$U_{c}=\left\{u_{1},\ldots ,u_{n_{c}}\right\}$	C’s user set
$U_{s}=\left\{v_{1},\ldots ,v_{n_{s}}\right\}$	S’s user set
*I*	the intersection of $U_{c}$ and $U_{s}$
*F*	facility location set
*L*	user location set
*Q* (or Q)	the desired query result
$n_{s}$, $n_{c}$, $n_{I}$ and *k*	the cardinalities of the sets $U_{s}$, $U_{c}$, *I* and *F*
(sk,pk)	the key pair of Paillier cryptosystem
$label_{i}$	a label looks random
H()	a hash function
τ()	a random permutation

## Appendix A. Figures of Experimental Results

We give the experimental results (of Section 8.2) in Figure A1 and Figure A2.

## References

[1] Kahraman C., Ruan D., Doğan I. (2003). Fuzzy Group Decision-making for Facility Location Selection. Inf. Sci..

[2] Du Y., Zhang D., Xia T. The optimal-location query. Proceedings of the International Symposium on Spatial and Temporal Databases.

[3] Yilmaz E., Ferhatosmanoglu H., Ayday E., Aksoy R.C. (2017). Privacy-preserving aggregate queries for optimal location selection. IEEE Trans. Dependable Secur. Comput..

[4] Ion M., Kreuter B., Nergiz E., Patel S., Saxena S., Seth K., Shanahan D., Yung M. (2017). Private intersection-sum protocol with applications to attributing aggregate ad conversions. IACR Cryptol. ePrint Arch..

[5] Roussopoulos N., Kelley S., Vincent F. Nearest neighbor queries. Proceedings of the 1995 ACM SIGMOD International Conference on Management of Data.

[6] Korn F., Muthukrishnan S. (2000). Influence sets based on reverse nearest neighbor queries. ACM Sigmod Rec..

[7] Stanoi I., Agrewal D., Abbadi A.E. Reverse nearest neighbor queries for dynamic databases. Proceedings of the ACM SIGMOD Workshop on Research Issues in Data Mining and Knowledge Discovery.

[8] Tao Y., Papadias D., Lian X. Reverse kNN search in arbitraty dimensionality. Proceedings of the Very Large Data Bases Conference (VLDB).

[9] Yang C., Lin K.I. An index structure for efficient reverse nearest neighbor queries. Proceedings of the 17th International Conference on Data Engineering.

[10] Stanoi I., Riedewald M., Agrawal D., Abbadi A.E. Discovery of influence sets in frequently updated databases. Proceedings of the 27th International Conference on Very Large Data Bases.

[11] Zhang D., Du Y., Xia T., Tao Y. Progressive computation of the min-dist optimal-location query. Proceedings of the 32nd International Conference on Very Large Data Bases.

[12] Qi J., Zhang R., Wang Y., Xue A.Y., Yu G., Kulik L. (2014). The min-dist location selection and facility replacement queries. World Wide Web.

[13] Mitra S., Saraf P., Bhattacharya A. (2019). Tips: Mining top-k locations to minimize user-inconvenience for trajectory-aware services. IEEE Trans. Knowl. Data Eng..

[14] Islam M.S., Shen B., Wang C., Taniar D., Wang J. (2020). Efficient processing of reverse nearest neighborhood queries in spatial databases. Inf. Syst..

[15] Du W., Atallah M.J. Secure multi-party computation problems and their applications: A review and open problems. Proceedings of the 2001 Workshop on New Security Paradigms.

[16] Du W., Atallah M.J. (2001). Protocols for secure remote database access with approximate matching. E-Commerce Security and Privacy.

[17] Hacıgümüş H., Iyer B., Mehrotra S. (2004). Efficient execution of aggregation queries over encypted databases. Proceedings of the International Conference on Database Systems for Advanced Applications.

[18] Cheng R., Zhang Y., Bertino E., Prabhakar S. (2006). Preserving user location privacy in mobile data management infrastructures. Privacy Enhancing Technologies.

[19] Mokbel M.F., Chow C.-Y., Aref W.G. The new casper: Query processing for location services without compromising privacy. Proceedings of the 32nd International Conference on Very Large Data Bases.

[20] Qi Y., Atallah M.J. Efficient privacy-preserving k-nearest neighbor search. Proceedings of the 2008 the 28th International Conference on Distributed Computing Systems.

[21] Thompson B., Haber S., Tomas H., Yao D. (2009). Privacy-preserving computation and verification of aggregate queries on outsourced databases. International Symposium on Privacy Enhancing Technologies Symposium.

[22] Yi X., Paulet R., Bertino E., Varadharajan V. Practical k nearest neighbor queries with location privacy. Proceedings of the 2014 IEEE 30th International Conference on Data Engineering.

[23] Chen H., Huang Z., Laine K., Rindal P. Labeled PSI from fully homomorphic encryption with malicious security. Proceedings of the 2018 ACM SIGSAC Conference on Computer and Communications Security.

[24] Paillier P. (1999). Public-key cryptosystems based on composite degree residuosity classes. Proceedings of the International Conference on the Theory and Applications of Cryptographic Techniques.

[25] Goldreich O. (2009). Foundations of Cryptopraphy: Volume 2.

[26] Lindell Y. (2017). How to simulate it-a tutorial on the simulation proof technique. Tutorial on the Foundations of Cryptography.

[27] Kissner L., Song D. (2005). Privacy-preserving set operations. Proceedings of the Annual International Cryptology Conference.

[28] Shekhar S., Kohli A., Coyle M. Path Computation Algorithms for Advanced Traveller Information System (ATIS). Proceedings of the IEEE 9th International Conference on Data Engineering.

[29] Damgård I., Jurik M., Nielsen J.B. (2010). A generalizetion of Paillier’s public-key system with applications to electronic voting. Int. J. Inf. Secur..

[30] Ge T., Zdonik S. Answering aggregation queries in a secure system model. Proceedings of the 33rd International Conference on Very Large Data Bases.

[31] Yang D., Zhang D., Zheng V.W., Yu Z. (2014). Modeling user activity preference by leveraging user spatial temporal characteristics in LBSNs. IEEE Trans.Syst. Man Cybern. Syst..

